# Small-RNA Deep Sequencing Reveals *Arctium tomentosum* as a Natural Host of *Alstroemeria virus X* and a New Putative Emaravirus

**DOI:** 10.1371/journal.pone.0042758

**Published:** 2012-08-17

**Authors:** Yaqi Bi, Arthur K. Tugume, Jari P. T. Valkonen

**Affiliations:** Department of Agricultural Sciences, University of Helsinki, Helsinki, Finland; University of Ottawa, Canada

## Abstract

**Background:**

*Arctium* species (Asteraceae) are distributed worldwide and are used as food and rich sources of secondary metabolites for the pharmaceutical industry, e.g., against avian influenza virus. RNA silencing is an antiviral defense mechanism that detects and destroys virus-derived double-stranded RNA, resulting in accumulation of virus-derived small RNAs (21–24 nucleotides) that can be used for generic detection of viruses by small-RNA deep sequencing (SRDS).

**Methodology/Principal Findings:**

SRDS was used to detect viruses in the biennial wild plant species *Arctium tomentosum* (woolly burdock; family Asteraceae) displaying virus-like symptoms of vein yellowing and leaf mosaic in southern Finland. Assembly of the small-RNA reads resulted in contigs homologous to *Alstroemeria virus X* (AlsVX), a positive/single-stranded RNA virus of genus *Potexvirus* (family Alphaflexiviridae), or related to negative/single-stranded RNA viruses of the genus *Emaravirus*. The coat protein gene of AlsVX was 81% and 89% identical to the two AlsVX isolates from Japan and Norway, respectively. The deduced, partial nucleocapsid protein amino acid sequence of the emara-like virus was only 78% or less identical to reported emaraviruses and showed no variability among the virus isolates characterized. This virus—tentatively named as Woolly burdock yellow vein virus—was exclusively associated with yellow vein and leaf mosaic symptoms in woolly burdock, whereas AlsVX was detected in only one of the 52 plants tested.

**Conclusions/Significance:**

These results provide novel information about natural virus infections in *Acrtium* species and reveal woolly burdock as the first natural host of AlsVX besides *Alstroemeria* (family Alstroemeriaceae). Results also revealed a new virus related to the recently emerged *Emaravirus* genus and demonstrated applicability of SRDS to detect negative-strand RNA viruses. SRDS potentiates virus surveys of wild plants, a research area underrepresented in plant virology, and helps reveal natural reservoirs of viruses that cause yield losses in cultivated plants.

## Introduction

Viruses occurring in communities of non-cultivated plants have gained limited attention as compared to those causing significant yield losses in cultivated plants [1]. However, wild plants can be natural reservoirs of new viruses that can be transmitted to crop plants. Perennial and biennial plant species are particularly potent virus reservoirs because they maintain viruses from one growing season to the next. On the other hand, worldwide transport of cultivated plants can disseminate viruses over long distances [2], [3] and causes a risk of introduction of viruses to new areas where some local wild species may become infected and serve as new virus reservoirs. For example, tens of native perennial or biennial wild species of genus *Ipomoea* (family Convolvulaceae) occur in Uganda where they are infected with the same viruses and virus strains as the cultivated sweetpotato (*Ipomoea batatas* Lam.) believed to have originated in Latin America. Some of the viruses were most likely introduced to Uganda with sweetpotatoes because those viruses are globally distributed in the crop, unlike some other viruses that seem to be of East African origin [3]–[6]. In Australia, the shrub-like perennial legume species *Hardenbergia comptoniana* (Andrews) Benth. (family Fabaceae) is a virus reservoir with demonstrated potential to act as a source of virus infection of the cultivated legume *Lupinus angustifolius* L. (family Fabaceae) [7]. In temperate regions, perennial grasses act as virus reservoirs that cause severe diseases such as yellow dwarf in cereals [8]–[10].

One reason for the limited number of studies addressing virus infections in wild plants is that such studies are particularly challenging. The serological and molecular methods routinely used for virus detection require at least some advance knowledge of the viruses expected in the samples so that appropriate antibodies, primers, or probes can be selected for use. When reasonable presumptions are lacking, virus detection becomes an arduous and time-consuming research process requiring many experiments and techniques, e.g., electron microscopy to observe virus particles, transmission of the virus to a range of indicator plants by mechanical or graft-inoculation to observe infectivity and symptoms, isolation of double-stranded RNA (dsRNA) for characterization of viral genome fragments, *etc.* [11]. The task is further complicated because plants in the field often have mixed virus infections. Finally, viruses causing no apparent symptoms in their wild host are likely to remain unattended.

A recently developed, novel molecular method (small-RNA deep-sequencing, SRDS) provides an effective solution to the aforementioned challenges in virus detection. It is a generic method that allows detection of both RNA and DNA viruses, provided that their genomes contain regions 30–40% identical to previously described viruses [12]. SRDS-based virus detection takes advantage of the natural, cellular antiviral defense mechanism called RNA silencing, which is induced by dsRNA. The dsRNA is detected by the dsRNA-specific cellular Dicer-like endonucleases (DCLs) and then degraded to fragments of 21–24 nucleotides (nt), designated as small interfering RNA (siRNA) [13]–[15]. The mechanism detects viruses because the single-stranded RNA (ssRNA) viruses form double-stranded intermediates during replication. Furthermore, incomplete or non-polyadenylated copies of the viral genomes or gene transcripts may be detected by a cellular RNA-dependent RNA polymerase, which synthesizes a complementary strand and generates dsRNA. Viral genomes contain ‘hotspots’ from which virus-derived siRNAs (vsiRNAs) are abundantly generated [16]. It is unclear, however, why certain viral genomic regions produce so many vsiRNAs. They do not seem to correspond to regions in which the viral genomic RNA or viral gene transcripts form loop-like dsRNA structures that can be targeted by the host silencing mechanism [14]. The role of siRNA molecules in RNA silencing is to guide the RNA silencing complex, which contains the ssRNA-specific ribonuclease Argonaute 1 (AGO1), to target, cleave, and inactivate the ssRNA that gave rise to the dsRNA and was detected by DCL. In antiviral defense, the target of the RNA silencing complex is the ssRNA genome of the virus, or a viral gene transcript. A secondary pool of vsiRNA is generated by the cellular RNA-dependent RNA polymerases RDR1 and RDR6 during the amplification phase of RNA silencing [15], which adds significantly to the vsiRNA levels in cells and also allows systemic signaling for antiviral defense in plants [17].

RNA silencing is seemingly always activated in virus-infected plants, also in those that display disease symptoms. The reason why viruses can multiply and spread in plants despite RNA silencing is that virus genomes encode proteins that partially suppress the silencing [18]. Hence, high accumulation of viral RNA and vsiRNAs are correlated in virus diseased, susceptible plants that cannot recover from virus infection [19]. For virus detection, the small-RNA pool can be isolated from the plant and subjected to deep sequencing. The small-RNA reads are assembled to longer contiguous sequences (contigs) and used as queries in sequence databases to identify homologous viral sequences [12].

The first study to utilize SRDS to detect unknown viruses identified three new ssDNA viruses belonging to the families *Caulimoviridae* and *Geminiviridae* in sweetpotato [12]. The analyzed plants had been inoculated with two positive-strand ssRNA [(+)ssRNA] viruses, *Sweet potato feathery mottle virus* (genus *Potyvirus*; Potyviridae) and *Sweet potato chlorotic stunt virus* (genus *Crinivirus*; Closteroviridae), and high numbers of vsiRNAs were detected [12]. More recently, SRDS was used to detect (+)ssRNA viruses of the genera *Foveavirus* (family Betaflexiviridae), *Maculavirus*, *Marafivirus* (family Tymoviridae), and *Nepovirus* (family Secoviridae) in grapevine plants (*Vitis vinifera* L.; Vitaceae) infected in the field [20], which showed that a severe virus disease in grapevines in the Midwestern USA was associated with badnaviruses (family Caulimoviridae) [21]. SRDS was also used to detect *Cereal yellow dwarf virus* (genus *Luteovirus*; Luteoviridae) in wild cocksfoot grass (*Dactylis glomerata* L.; Poaceae) in the United Kingdom [10]. These studies demonstrate the applicability of SRDS for detection of many unrelated viruses in a wide taxonomic range of plants.

Burdock plants (*Arctium* spp., family Asteraceae) are rich sources of pharmaceutical compounds. Arctiin and its aglucone obtained from the tap roots of burdock can interfere with early stages of replication of the avian influenza virus and can also hamper progeny virus release in mammalian cells. Therefore, these compounds are studied as alternative antiviral medicines against the virus strains resistant to oseltamivir (Tamiflu) [22], [23]. The tap roots of greater (edible) burdock (*A. lappa* L.) are consumed particularly in Asia [24]. Woolly burdock (*Arctium tomentosum* Mill.) is a wild species common throughout most parts of Europe, northern USA, Canada, and Asia [25]. It occupies temporary niches during succession from annual to perennial vegetation and is usually found at edges of fields, gardens, roadsides, fence lines, waste dumps, and other human-impacted environments [26]. Woolly burdock completes its life cycle biennially. In the first year, it produces a leaf rosette and a large fleshy tap root as the storage organ. In the second year, a rosette of large leaves, up to 45 cm long by 40 cm wide, is formed, with white and woolly hair beneath. Subsequently, a green, lignified, branched stem up to 2 m high with flower heads at the ends of branches is formed. After flowering, plants die and the seeds with a bristling hook structure are dispersed by attaching to animal skin, fur, human clothes, *etc.* [26].

In this study, our attention was drawn to the virus-like symptoms such as vein yellowing and mosaic symptoms in leaves that are common in the leaves of the second-year plants of woolly burdock in southern Finland. The symptoms are readily observed in young plants but the disease etiology is unknown. The aim of this study was to apply SRDS to detect possible viruses in woolly burdock plants displaying aforementioned symptoms.

## Materials and Methods

### Sample collection and plant species identification

Plants of *A. tomentosum*, representing the second-year, reproductive phase, were inspected in four areas of the Helsinki capitol area (Oulunkylä, Pasila, Pihlajamäki, and the Viikki Research Farm area) in Finland in May (2010) and June (2009 and 2010). The plants had not started flowering or were at an early stage of flowering at the time of inspection. Symptomless plants and plants displaying vein yellowing and mosaic symptoms in leaves were sampled at random. The leaf samples were put into liquid nitrogen immediately after collection and stored at −80°C until use for RNA extraction. The six symptomatic plants that were subjected to SRDS were sampled in Pihlajamäki in June 2009; all other samples were collected from the aforementioned four places in June 2010. No specific permits were required for the described field studies. The locations were not privately owned, and field studies did not involve endangered species.

The sampled plants were identified based on morphological characteristics [25], and a botanist confirmed each such identification. Furthermore, the maturase K (*matK*) gene sequence was determined and used as a plant “barcode" for comparison with other species [27]. It should be noted that *Arctium* species share similar growth habitats and can cross-breed, which may hamper morphology-based species identification of the plants. Total DNA was extracted from leaves of two plants using the cetyltrimethylammonium bromide method [28]. A pair of universal primers for land plants, matK2.1af (5′-ATCCATCTGGAAATCTTAGTTC-3′) and matK5r (5′- GTTCTAGCACAAGAAAGTCG-3′) as listed on Kew Royal Botanic Garden's DNA Barcoding website (http://www.kew.org/barcoding/protocols.html), was used to amplify the gene by polymerase chain reaction (PCR). PCR products of the expected size were purified from an agarose gel using E.Z.N.A. gel extraction kit D2500-02 (Omega Bio-Tek, Norcross, GA, USA) according to the manufacturer's instructions. DNA sequencing was done at Haartman Institute (University of Helsinki). Sequences were analyzed using the Basic Local Alignment Search Tool (BLAST) of the National Center for Biotechnology Information (NCBI). The *matK* sequences of the two plants were identical and showed the closest (98%) nucleotide identity to *matK* of *Arctium lappa* L. (NCBI sequence accession no. HM989769). These results supported morphological identification of the plants to the species *A. tomentosum* whose *matK* sequence was not available prior to our study. The sequence was submitted to the NCBI sequence database under the accession no. JQ354896.

### Total RNA extraction and small-RNA deep sequencing

Total RNA was extracted from leaf samples (0.4 g) using Trizol reagent (Invitrogen, Carlsbad, CA, USA). The concentration and purity of RNA samples were determined with a spectrophotometer. Agarose gel electrophoresis was used to check integrity of the high molecular weight RNA.

For SRDS, equal amounts of total RNA from six symptomatic *A. tomentosum* plants were pooled, and 10 µg from the RNA pool was sequenced by Fasteris SA (Plan-les-Ouates, Switzerland). The main steps of SRDS included separation of RNA by polyacrylamide gel electrophoresis, isolation of small RNA (<30 nt) from the gel, and ligation with a single-stranded 3′-adapter and a bar-coded 5′-adapter. Ligated RNA was reverse transcribed and amplified by PCR to generate a DNA colony template library, which was purified and diluted to a concentration of 10 nM. An Illumina Genome Analyzer was used for high-throughput DNA sequencing.

### Sequence analysis

The program Velvet [29] was used for *de novo* assembly of siRNA reads as in previous studies [10], [12] to produce contigs by assembly of small-RNA reads based on De Bruijn graphs, which are data elements organized around words of *k* nucleotides (*k*-mer) [29]. Reads were hashed according to a predefined *k*-mer (number of nucleotides overlapping between reads), which was varied during the analysis because a larger *k*-mer brings out more specific contigs but decreases the read coverage whereas a small *k*-mer produces contigs with high coverage but with lower specificity. The error-correction functions of Velvet were utilized [29]. Because the concentrations of some viruses and the vsiRNAs might be relatively low in the host plant, the coverage cutoff was reduced (set to 5) to prevent loss of small amounts of viral reads. The contigs assembled by Velvet were used to search similar sequences in databases using BLAST. The “Organism" option of BLAST was set to detect only viruses to prevent mismatches, especially for short contigs.

When viral sequences had been identified among the contigs, complete sequences of the most similar viruses were retrieved from sequence databases and used as references for alignment of small-RNA reads with MAQ (MAQ User's Manual, release 0.5.0) as in previous studies [12], [21] and also Novoalign. The programs are similar, but Novoalign optimizes the algorithm to produce a more accurate alignment score. Both programs were used to build assemblies by mapping shotgun reads to reference virus sequences [30]. Eventually, all viral genome data available in the database were used as a reference to increase the chance to detect novel viruses, i.e., because there was no prior information about viruses occurring in *A. tomentosum*. MultAlin and ClustalW2 were used to align multiple sequences and analyze coverage quality.

### Virus detection by RT-PCR

The viruses detected by SRDS were characterized further by amplification of partial genome sequences using reverse transcription PCR (RT-PCR). cDNA was synthesized using an Oligo(T)23 primer or random hexamer primers (Oligomer Oy, Helsinki, Finland). The RT reaction containing 0.1–02 µg of total RNA and *Moloney murine leukemia virus* (M-MLV) reverse transcriptase (Promega, Madison, WI, USA), following the manufacturer's instructions. cDNA was diluted 2-fold and stored at −20°C until use.

Primers were designed with the program Primer3 (http://simgene.com/Primer3) to amplify a 3′-proximal region, including the coat protein (CP) gene of *Alstroemeria virus X* (AlsVX) and the putative nucleocapsid protein (NP) gene of a Fig mosaic-like virus detected in this study (tentatively designated as Woolly burdock yellow vein virus, WBYVV). Nested PCR was used to amplify AlsVX. For the first PCR, the forward primer (5′-CCACCGCCTAACTACGAAAA-3′, nt 5761–5780) was designed according to the isolate AlsVX-Jap described in Japan [31]. The Oligo(T)23 primer was used as the reverse primer. The reaction mix contained 2.5 µl of 10× Dynazyme DNA polymerase buffer (Finnzymes), 0.3 µl 10 mM dNTPs, 0.7 µl of the primers (10 µM final concentration), 0.3 µl (1 U) Dynazyme high-fidelity DNA polymerase, 2.5 µl cDNA, and 18 µl of sterile deionized water. The PCR program was 3 min at 94°C, 5 cycles of 30 sec at 94°C, 30 sec at 50°C, and 75 sec at 72°C, followed by 30 cycles of 30 sec at 94°C, 30 sec at 52°C, and 75 sec at 72°C, and final extension for 10 min at 72°C. The expected product size was 1348 nt [31]. In the second round of PCR, the reaction mix was as above except that 2.5 µl of the PCR product from the first PCR was used as the template. The primers were designed according to AlsVX-Jap except that a few nucleotide substitutions (underlined here) were introduced to the primer-binding sites based on the MAQ mapping of vsiRNA reads: 5′-GGCGACCAACAACATTCTCT-3′ (forward primer, nt 5860–5879) and 5′-CAAACTGTAGAAAAACACCTCC-3′ (reverse primer, nt 6960–6981). The PCR program differed from the first PCR, as follows: denaturation 3 min at 94°C, 35 cycles of 30 sec at 94°C, 30 sec at 56°C, and 75 sec at 72°C, followed by final extension for 10 min at 72°C. The expected size of the PCR product was 1120 nt.

To detect WBYVV, the PCR reactions and cycling were similar to those used in the first PCR for AlsVX amplification, except that the 30 cycles of PCR were as follows: 30 sec at 94°C, 30 sec at 53°C, and 75 sec at 72°C. The forward (5′-TCAACAAACGACTTCCTCACTG-3′) and reverse (5′-AGGGTGCTGATCTGTCTGCT -3′) primers were designed according to the contigs built by Velvet and, based on comparison to FMV sequences, were expected to amplify a 604-nt region of the NP gene. PCR products were purified and sequenced at Haartman Institute as described above. Sequences were analyzed with ClustalW2 and BLAST. All new sequence data were deposited in the NCBI sequence database.

### Phylogenetic analysis

Nucleotide sequences of emaraviruses were translated into amino acid sequences and these alignments were used to facilitate alignment of nucleotide sequences of different viruses and virus isolates using ClustalW. The viral sequences retrieved from databases were trimmed to the same length with the NP gene sequence of WBYVV determined in this study. Phylogenetic trees were constructed using the neighbor-joining algorithm [32] implemented in MEGA4 [33] using the Kimura two-parameter nucleotide substitution model [34] and the default bootstrap option.

## Results

### SRDS reveals potexvirus and emaravirus infection in *A. tomentosum*

Velvet could assemble 97 and 75 contigs in the size range of 100–299 nt using the *k*-mer values 15 and 17, respectively. Seven contigs of 300–1000 nt were obtained with either *k*-mer value. When subjected to BLAST, many contigs showed high identity to FMV, *Fig mosaic-associated virus* (FMAV), and *European mountain ash ringspot-associated virus* (EMARAV) representing multipartite, negative-strand ssRNA [(−)ssRNA] viruses that belong to the genus *Emaravirus* (not assigned to any family) [35]. Furthermore, many contigs showed high identity to monopartite (+)ssRNA viruses of the genus *Potexvirus* (family *Alphaflexiviridae*) [35], especially to AlsVX.

A similar number of vsiRNA reads (21–24 nt) were mapped to the viral genomes (Table 1) using MAQ and Novoalign (Table 2). The greatest number of vsiRNAs mapped to the AlsVX genome regardless of vsiRNA length, and 37% of the genome sequence of AlsVX [31] was covered. The next highest number of reads (15–17% genome coverage) was mapped to FMAV and FMV sequences (Table 2). Among the total number of 3,758,846 reads in the size range 21–24 nt, Novoalign could map 6940 reads (0.18%) and 1958 reads (0.05%) to the AlsVX and FMAV genomes, respectively. Among the 21-nt reads (n = 603,783), 3167 (0.52%) and 1271 (0.21%) reads were mapped to AlsVX and FMAV, respectively; among the 22-nt reads (n = 341,407), 2393 reads (0.70%) and 344 reads (0.10%) were mapped, respectively.

**Table 1:** Viral sequences obtained from public databases and used in the study.

Virus	Acronym	Sequence accession no.
Genus *Potexvirus*:		
*Alstroemeria virus X*, Finland^a^	AlsVX-Hel	JQ354895
*Alstroemeria virus X*, Japan	AlsVX-Jap	AB206396
*Alstroemeria virus X*, Norway	AlsVX-HelE91	JN820315.1
*Asparagus virus 3*	AV-3	AB304848
*Chenopodium mosaic virus X*	ChMVX	DQ660333
*Lettuce virus X*	LeVX	AM745758
*Narcissus mosaic virus*	NMV	AY225449
*Pepino mosaic virus*	PepMV	AY509926
*Scallion virus X*	ScaVX	AJ316085
Genus *Emaravirus* b		
*European mountain ash ringspot-associated virus*	EMARAV	GU563319
*Fig mosaic virus*, isolate Can01	FMV-Can01	HQ703345
*Fig mosaic virus*, isolate Gr10	FMV-Gr10	FM991954
Fig mosaic-associated virus	FMAV	FJ211073
*Maize red stripe virus*	MRSV	DQ324466
*Raspberry leaf blotch virus*	RLBV	FR823301
*Rose rosette virus*	RRV	HQ891906
Woolly burdock yellow vein virus^a^	WBYVV	JQ354894

a The CP (AlsVX) or partial NP gene sequence (WBYVV) was determined in this study by amplification of the viral genomic region using RT-PCR and sequencing of the products.

b Established and putative members of the genus.

**Table 2:** Numbers of vsiRNA reads mapped to reference virus genomes by MAQ and Novoalign.

	21-nt reads		22-nt reads		23-nt reads		24-nt reads	
	n = 603783^a^		n = 341407		n = 373102		n = 2440554	
Virus	MAQ	NOVO	MAQ	NOVO	MAQ	NOVO	MAQ	NOVO
								
AlsVX	3 142	3 167	2 304	2 393	386	460	731	920
FMAV	1 261	1 271	340	344	109	111	209	232
FMV	1 074	1 105	318	341	72	85	143	198
AV3	668	687	386	409	62	72	159	221
LeVX	511	525	348	366	67	74	61	123
ScaVX	411	420	256	270	42	48	75	127
EMARAV	377	391	148	151	20	29	46	67
NMV	366	380	229	251	45	52	60	137
CMVX	257	276	140	169	39	45	43	104
PeMV	141	141	49	61	10	20	26	51

a Total number of reads obtained by small-RNA deep sequencing.

In general, 45.6% and 64.9% of all the small-RNA reads that mapped to AlsVX and FMAV genomes, respectively, belonged to the 21-nt class. The 22-nt vsiRNAs were the next most abundant group (34.5% and 17.6%, respectively), followed by 24-nt (13.3% and 11.8%) and 23-nt (6.6% and 5.7%) vsiRNAs (Table 2).

Large numbers of vsiRNA reads could also be mapped to the genomes of seven additional viruses (Table 1): *Asparagus virus 3*, *Lettuce virus X*, *Scallion virus X*, *Narcissus mosaic virus*, *Chenopodium mosaic virus X* (also called Malva mosaic virus), and *Pepino mosaic virus* belonging to the genus *Potexvirus*, and EMARAV (genus *Emaravirus*) (Table 2). These matches were due to the regions of genomes conserved in the related viruses of *Potexvirus* and *Emaravirus*.

#### Characterization of the CP gene of AlsVX-Hel

Primers were designed to AlsVX genome regions flanking the CP gene that were covered by high numbers of vsiRNA reads (i.e., deeply sequenced regions). Subsequently, the region corresponding to nt 5880–6959 in AlsVX-Jap (genome length 7009 nt) [31] including the whole CP gene was amplified by nested RT-PCR from the RNA pool that had been used for SRDS. Each of the six samples included in the RNA pool was also tested separately. Only one of them (Pe15) was AlsVX-positive. The PCR product was sequenced (GenBank accession no. JQ354895) and compared with the CP sequence of AlsVX-Jap [31] and the very recently published CP gene sequence of AlsVX-HelE91 from Norway (accession no. JN820315), indicating 81% and 89% nucleotide identity, respectively. Identities of the deduced amino acid sequences between the isolate detected in this study (designated as AlsVX-Hel) and the other two isolates were 90% and 98%, respectively (Table 3). According to BLASTx results, the CP sequence of AlsVX-Hel also showed lower but significant identity with many other potexviruses (Table 3).

**Table 3:** Percentage nucleotide (upper triangle) and deduced amino acid identities (lower triangle) of the coat protein genes of three isolates of *Alstroemeria virus X* and other potexviruses (see Table 1 for sequence accession numbers).

	AlsVX -Hel	AlsVX -Jap	AlsVX -HelE91	NMV	ScaVX	AV3	CMVX	PeMV	LeVX
AlsVX-Hel	*	81	89	70	69	71	64	63	67
AlsVX-Jap	90	*	82	69	68	68	65	64	70
AlsVX-HelE91	98	91	*	69	71	70	65	63	67
NMV	73	74	72	*	72	74	71	63	68
ScaVX	68	67	68	78	*	82	70	63	66
AV3	69	68	68	83	87	*	70	64	67
CMVX	66	65	65	77	72	75	*	64	61
PeMV	64	64	64	67	63	64	59	*	61
LeVX	64	64	63	67	61	60	57	58	*

#### Characterization of partial NP gene sequence of WBYVV

Following MAQ-based mapping of vsiRNA reads to the FMV genome sequence, primers were designed according to those terminal regions of RNA3 that were covered by the highest numbers of vsiRNA reads. Subsequently, a region of the NP gene corresponding to nt 487–972 in the RNA3 of FMV-Gr10 [36], [37] was amplified by RT-PCR. The six plants of *A. tomentosum* included in the pool of RNA subjected to SRDS were all PCR-positive, and the sequences obtained were identical (NCBI accession no. JQ354894). The virus was designated tentatively as WBYVV, because the partial NP sequence showed only 75–78% nucleotide and amino acid sequence identity with previously described FMV isolates (Gr10 and Can01) and FMAV. The corresponding regions in FMV-Gr10, FMV-Can01, and FMAV were 94–98% and 99–100% identical at the nucleotide and amino acids levels, respectively (Table 4). The nucleotide and amino acid sequences of *Rose rosette virus* (RRV) were 69% and 74% identical, respectively, to WBYVV (Table 4). Hence, WBYVV could not be readily equated with any of these emaraviruses. The nucleotide and amino acids sequences of EMARAV were 59% and 52%, respectively, identical to WBYVV, and those of Raspberry leaf blotch virus (RLBV) were 47% and 10% identical, respectively (Table 4).

**Table 4:** Percentage nucleotide (518 nt; upper triangle) and deduced amino acid identities (lower triangle) of the nucleocapsid protein gene of WBYVV and related negative-strand ssRNA viruses (see Table 1 for sequence accession numbers).

	WBYVV	FMV-Can01	FMV-Gr10	FMAV-JJW2008	RRV	EMARAV	MRSV	RLBV
WBYVV	*	78	77	78	69	59	50	47
FMV- Can01	78	*	94	98	70	66	55	56
FMV-Gr10	78	100	*	94	69	64	56	56
FMAV-JJW2008	75	99	99	*	70	63	45	46
RRV	74	65	62	75	*	58	57	59
EMARAV	52	41	41	50	42	*	56	53
MRSV	27	24	24	25	23	20	*	55
RLBV	10	15	15	9	10	10	10	*

**Figure 1:**
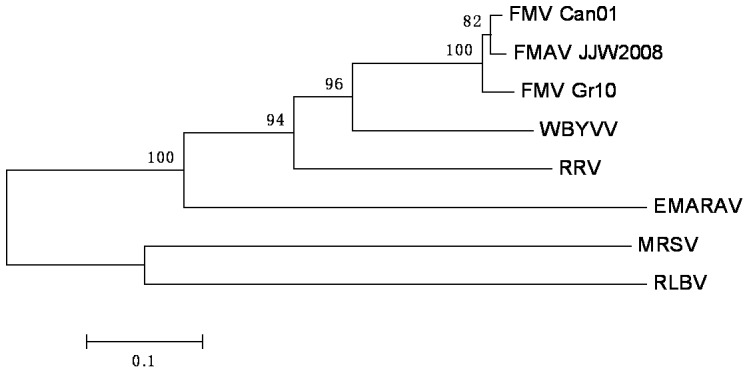
Phylogenetic analysis of partial putative nucleocapsid protein (NP) gene sequences (518 nt; RNA3) of established and putative members of genus *Emaravirus*. The NP gene sequence of *Pigeonpea sterility mosaic virus* is not available and could not be included. Bootstrap values higher than 80 (of 100 replicates) are indicated on the branches. For accession numbers, see Table 1. Bar indicates 0.1 Kimura units [34].

Phylogenetic analysis of the partial NP gene sequences indicated that WBYVV is only distantly related to the FMV strains from Canada (FMV-Can01) and Italy (FMV-Gr10) [26], [37] and to FMAV from USA [38] or to RRV [39] and EMARAV [40]. Moreover, there is even further evolutionary distance between WBYVV and *Maize red stripe virus* (MRSV) [41] and RLBV [42], which form a phylogenetic cluster different from other emaraviruses (Fig. 1).

### Distribution and variability of AlsVX and WBYVV in *A. tomentosum*

**Figure 2:**
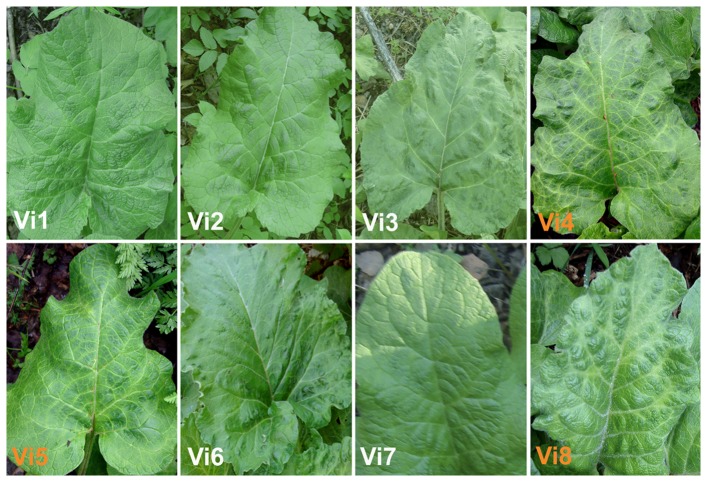
Leaves of woolly burdock (*A. tomentosum*) plants with and without virus-like symptoms. Plants Vi4, Vi5 and Vi8 (orange labels) were infected with WBYVV and the leaves display vein yellowing and mosaic symptoms. Plants were photographed in the Viikki Research Farm area on May 18, 2011.

The six plants of WBYVV-infected *A. tomentosum* (one plant also infected with AlsVX-Hel) tested by SRDS in 2009 grew within a distance of 50 m from each other at the edge of a pedestrian pathway. The same and three additional locations 1–10 km apart from each other were surveyed for virus-like symptoms in *A. tomentosum* in the next year. In all locations, most *A. tomentosum* plants displayed virus-like symptoms, such as vein yellowing, mild mosaic and epinasty in leaves (Fig. 2). A total of 41 plants (79%) of the randomly sampled 52 plants from all sampling sites were positive for WBYVV, whereas no plant was positive for AlsVX, as tested by RT-PCR using primers designed for the respective viruses. All PCR-positive plants showed the aforementioned symptoms, in contrast to PCR-negative plants, which were symptomless (Fig. 2).

PCR products were sequenced from two plants per sampling site and found to be identical. The analysis was repeated with new samples and reagents and negative controls with similar results. Hence, the characterized region of the NP gene in WBYVV isolates showed no detectable sequence variability.

## Discussion

AlsVX was originally detected in *Alstroemeria* (lily of the Incas; family Alstroemeriaceae) in Japan where it co-infects this ornamental plant with other viruses [31]. *Alstroemeria* forms rhizomes and storage roots, which are used in vegetative propagation of plants. The infected propagules disseminate AlsVX and other viruses to new plants of *Alstroemeria* [43], [44]. AlsVX infection is difficult to detect in *Alstroemeria* because the virus is symptomless in this host [31]. However, AlsVX causes many types of symptoms in a broad range of other, experimentally infected hosts in seven additional plant families, namely Aizoaceae, Amaranthaceae, Asteraceae, Chenopodiaceae, Cucurbitaceae, Leguminosae, and Pedallaceae [31].

Our study reveals *Arctium* as a new natural host for AlsVX. Previously, AlsVX was known to naturally infect only *Alstroemeria* plants in Japan and has been recently reported in Norway [45] in another ornamental, strawflower [*Helichrysum bracteatum* (Vent.) Andrews, synonymous to *Xerochrysum bracteatum* (Vent.) Tzvelev] that also belongs to family Asteraceae [46]. The CP gene nucleotide sequences of the three currently known AlsVX isolates are only 81–89% identical, indicating significant genetic variability of the virus. *Alstroemeria* originated in South America and is a popular ornamental plant grown in most parts of the world. Whether AlsVX evolved in *Alstroemeria* or in other species remains to be studied. Similarly, the origin of AlsVX found in a wild plant of *A. tomentosum* in this study remains enigmatic. These issues could be addressed in future studies by using SRDS to detect and characterize more isolates of AlsVX in other plants and countries.

Biennial growth [26] and the typical habitats at edges of arable fields and gardens [47] make woolly burdock a highly potent reservoir of viruses of cultivated plants. Viruses survive in the tap root of woolly burdock until the next cropping or growing season, and close proximity to crop plants facilitates virus transmission by contact and vectors. Apart from our present study, there is only limited information about viruses occurring in *Arctium* species, but common (lesser) burdock [*Arctium minus* (Hill.) Bernh.] is a natural host of *Iris yellow spot virus* (IYSV, genus *Tospovirus*; Bunyaviridae) in the state of New York [48]. The virus causes significant yield losses in onion (*Allium cepa* L.; Amaryllidaceae) in the western USA and occurs in many annual and perennial weeds and crop plants in the field in various other parts of the USA. Common burdock is also a host for onion thrips (*Thrips tabaci* Lindeman), the vector of IYSV [49].

The majority of the woolly burdock plants we tested were naturally infected with WBYVV. The (−)ssRNA emaraviruses have not been assigned to any viral family, and in contrast to IYSV belonging to family *Bunyaviridae* [42] they are not transmitted by thrips but eriophyid mites, as reported for FMV [36], [37], [50], *Pigeon pea sterility mosaic virus* [51], EMARAV [52], and RLBV [42]. WBYVV was most closely related to FMV, but the partial NP gene characterized from the virus differs substantially from FMV and other emaraviruses [42], [53], and thus WBYVV cannot be equated unequivocally with any existing emaravirus species but will probably be assigned as a new virus species when the viral genome sequence is fully characterized. Hence, WBYVV may represent an additional member of the recently emerged group of (−)ssRNA plant viruses (genus *Emaravirus*). Our results show that (−)ssRNA viruses can be readily detected by SRDS.

The partial NP gene sequences determined from eight isolates of WBYVV were identical, although the isolates were collected from different locations up to 10 km apart. Similarly, only limited genetic variability was observed among the NP gene sequences of 20 isolates of EMARAV characterized from mountain ash trees (*Sorbus aucuparia* L.; Rosaceae) distributed over a much larger area of Finland and Russia (500×500 km). The genes were 97–99% identical and found to be under strong purifying selection [54], [55]. Some variability has been detected within a short NP gene fragment (298 nt) characterized from FMV isolates in Turkey [56]. In general, little is known about the intraspecific genetic variability of emaraviruses, and hence it remains unclear whether the limited variability of NP genes is representative of the level of variability of other parts of the genome.

Plants encode four DCLs (DCL1 to DCL4) with partially complementary or redundant activities [57]. DCL4 is the primary sensor of viral dsRNA and produces 21-nt vsiRNAs needed for antiviral defense [17], [58]. DCL2 produces 22-nt or 23-nt vsiRNAs [59], but only the 22-nt vsiRNAs are involved in antiviral defense [58]. The vsiRNAs from AlsVX and WBYVV determined in this study were proportionally most abundant in the small-RNA classes of 21 nt and 22 nt, as expected. Also, 23-nt vsiRNAs from both viruses were detected but in considerably lower numbers. DCL3 generates endogenous 24-nt siRNAs, which constitute the most abundant siRNA class in flowering plants [60]. DCL3 can also target viral dsRNA, as indicated by hundreds of 24-nt vsiRNAs from AlsVX and WBYVV detected by SRDS. However, neither the 24-nt nor 21-nt vsiRNAs generated by DCL1 are involved in antiviral defense [58]. The task of DCL1 is to generate 21-nt microRNAs that post-transcriptionally regulate plant gene expression [60].

Our study provides further evidence that SRDS is a powerful method for plant virus diagnostics, and also suitable for screening plants for known or new (−)ssRNA viruses. In a previous study SRDS was used to analyze vsiRNA profiles in plants experimentally inoculated with *Tomato spotted wilt virus*, a (−)ssRNA virus from genus *Tospovirus* (Bunyaviridae) [61]. The wide applicability of SRDS has been demonstrated in studies on invertebrates and mammalians, as shown by detection and characterization of RNA and DNA viruses infecting mosquitoes and flies [62], [63] and detection of *Human immunodeficiency virus 1* in a mammalian cell line [64]. For viruses having low titers in hosts, more sensitive detection could be achieved by subtracting the excess amounts of host-derived small-RNAs using the host genome sequence as a reference [64]. This approach will become more feasible as additional plant genome sequences become available. As shown by our study and previous work [10], [12], however, availability of the host genome sequence is not necessary for use of SRDS as a tool in virus diagnostics and few hundred to thousand 21-nt and/or 22-nt vsiRNA reads (less than 1% in the total small-RNA pool) are sufficient to detect known and related new viruses.

### Sequence Data

JQ354896 (*matK* gene of *A. tomentosum*), JQ354895 (CP gene sequence of AlsVX-Hel), JQ354894 (partial NP gene sequence of WBYVV).
